# Fenofibrate-associated rhabdomyolysis complicated by dialysis-requiring acute kidney injury presenting with atypical clinical manifestations: A case report and literature review

**DOI:** 10.1097/MD.0000000000050337

**Published:** 2026-08-21

**Authors:** Yumei Fang, Yan Jiang

**Affiliations:** a Department of Nephrology, The First People’s Hospital of Lin’an District, Hangzhou, Zhejiang, China.

**Keywords:** acute kidney injury, case report, chronic kidney disease, fenofibrate, hemodialysis, rhabdomyolysis

## Abstract

**Rationale:**

Rhabdomyolysis (RM) may cause acute kidney injury (AKI), but the classic triad of myalgia, muscle weakness, and dark-colored urine is often absent. Fenofibrate-associated RM is rare and may be overlooked in older patients with chronic kidney disease (CKD), reduced renal reserve, or polypharmacy.

**Patient concerns:**

A 70-year-old man with CKD was admitted with nausea, poor appetite, and a marked increase in serum creatinine for 1 week. Two months earlier, atorvastatin had been discontinued, and micronized fenofibrate had been started for hypertriglyceridemia. He had no obvious myalgia, muscle weakness, or dark-colored urine.

**Diagnoses:**

Serum creatinine rapidly increased from approximately 130 μmol/L to 1132–1202 μmol/L. Peak creatine kinase (CK) was 28,831 U/L, and serum myoglobin was 915 ng/mL, accompanied by metabolic acidosis, hypocalcemia, and hyperphosphatemia. Based on the temporal association with fenofibrate exposure, marked muscle enzyme elevation, improvement after drug withdrawal, and absence of common alternative triggers, the patient was diagnosed with probable fenofibrate-associated rhabdomyolysis complicated by KDIGO stage 3 AKI superimposed on underlying CKD.

**Interventions:**

Fenofibrate was discontinued immediately. The patient received fluid therapy, correction of metabolic acidosis, electrolyte monitoring and management, and supportive kidney care. Because he initially refused dialysis, intermittent hemodialysis (IHD) was initiated on hospital day 10 for persistent uremic symptoms, severe AKI, and metabolic derangements. Net ultrafiltration was set at 0 mL to avoid further volume depletion.

**Outcomes:**

After IHD and supportive treatment, gastrointestinal symptoms improved, urine output increased, and CK and renal function gradually improved. Dialysis frequency was reduced after discharge, and IHD was discontinued 5 weeks later. At 8 months after discharge, serum creatinine had decreased to 157 μmol/L, and CK and myoglobin remained normal.

**Lessons:**

In older patients with CKD, eGFR should be calculated before fenofibrate is prescribed, and dosing should follow the product label. Unexplained renal deterioration or nonspecific symptoms after fenofibrate exposure should prompt measurement of CK and myoglobin. Immediate drug withdrawal and supportive treatment are essential, whereas IHD should be reserved for standard AKI indications.

## 1. Introduction

Rhabdomyolysis (RM) is caused by skeletal muscle injury with release of intracellular contents, including creatine kinase (CK), myoglobin, electrolytes, and other cellular components, into the circulation.^[1,2]^ Its manifestations range from asymptomatic muscle enzyme elevation to electrolyte disturbances, arrhythmia, disseminated intravascular coagulation, and acute kidney injury (AKI), which is mediated mainly by myoglobin-related tubular toxicity, renal vasoconstriction, and intratubular cast formation.^[1-3]^

The causes of RM include trauma, crush injury, strenuous exercise, heat stroke, infection, metabolic or endocrine disorders, alcohol or toxin exposure, and drugs. Lipid-lowering drug-associated RM is most commonly associated with statins and may also occur with combined statin-fibrate therapy. Fenofibrate monotherapy-induced RM is rarely reported; however, the risk is increased in patients with advanced age, renal impairment, hypothyroidism, diabetes mellitus, or polypharmacy.^[4]^

Older patients often present with nonspecific symptoms such as fatigue, anorexia, nausea, vomiting, or oliguria and may lack typical myalgia, muscle weakness, and dark-colored urine. Such presentations may be misinterpreted as the progression of CKD or gastrointestinal disease. We report a case of suspected fenofibrate-associated RM complicated by dialysis-requiring AKI in an older man with CKD and a solitary kidney. The patient presented atypically with gastrointestinal symptoms and a rapid rise in serum creatinine and eventually discontinued hemodialysis after drug withdrawal and supportive treatment.

## 2. Case report

### 2.1. Patient information and clinical findings

A 70-year-old man was admitted to the Department of Nephrology on May 26, 2025, because of “elevated serum creatinine for more than 7 years and nausea with poor appetite for 1 week” (Table 1). Seven years previously, a routine physical examination had revealed an elevated serum creatinine level of approximately 100 μmol/L. Thereafter, he was followed regularly and had received unspecified traditional Chinese medicine. Two months before admission, his serum creatinine had remained approximately 130 μmol/L.

**Table 1:** Clinical timeline.

Time	Events/findings	Clinical significance
>7 yr before admission	Serum creatinine first noted to be elevated, approximately 100 μmol/L; regular follow-up thereafter	Underlying CKD
4 yr before admission	Right nephrectomy for right renal atrophy and abscess	Reduced renal reserve and increased AKI risk
>2 mo before admission	Serum creatinine approximately 130 μmol/L; atorvastatin replaced with fenofibrate because of elevated TG	Clear drug-exposure window
1 wk before admission	Fatigue, dizziness, poor appetite, nausea, and vomiting; no myalgia or dark urine	Atypical clinical presentation
Admission/day 2	Serum creatinine 1132–1202 μmol/L; CK 28,831 U/L; myoglobin 915 ng/mL; metabolic acidosis	Diagnosis of RM complicated by severe AKI
Hospital day 2	Fenofibrate discontinued; fluid therapy, correction of acidosis, and electrolyte monitoring started	Removal of suspected causative drug and initiation of supportive care
Hospital day 7	Serum creatinine 1218 μmol/L; CK 11,194 U/L; myoglobin 891 ng/mL	Muscle enzymes decreased, but renal function had not clearly recovered
Hospital day 10	Hemodialysis started 3 times weekly with net ultrafiltration set at 0 mL	Treatment of uremic symptoms and metabolic derangements while avoiding volume depletion
Hospital days 15–20	Serum creatinine 733→694 μmol/L; CK decreased to 228→98 U/L; urine output increased	Muscle injury controlled and renal function gradually improved
Weeks 1–6 after discharge	Serum creatinine 497→297 μmol/L, myoglobin 132 ng/mL, CK 72 U/L; dialysis gradually discontinued	Favorable clinical course
Months 3–8 after discharge	Serum creatinine 204→157 μmol/L	Sustained renal recovery

AKI = acute kidney injury, CK = creatine kinase, CKD = chronic kidney disease, RM = rhabdomyolysis, TG = triglyceride.

One week before admission, he developed generalized fatigue, dizziness, anorexia, and poor appetite, with food intake reduced to approximately one-third of his usual level. He also had nausea and vomiting of clear fluid 2 to 3 times per day. He denied fever, heat stroke, seizures, strenuous exercise, crush injury, muscle spasms, marked oliguria, gross hematuria, dark-colored urine, myalgia, or muscle tenderness.

His medical history included hypertension for more than 10 years, treated with losartan potassium 100 mg once daily and amlodipine besylate 5 mg once daily; cerebral infarction for more than 7 years, treated with clopidogrel hydrogen sulfate 75 mg once daily; hyperlipidemia for more than 10 years; chronic hepatitis B for several decades, treated with tenofovir disoproxil fumarate 0.3 g nightly; the most recent hepatitis B virus DNA level was within the reference range; a history of bladder cancer surgery more than 5 years previously; and right nephrectomy 4 years previously for right renal abscess with renal atrophy. He had previously taken atorvastatin. More than 2 months before admission, atorvastatin was replaced with micronized fenofibrate capsules (0.2 g once daily; Astrea Fontaine) because of elevated triglyceride levels despite low total and low-density lipoprotein cholesterol levels.

On admission, his temperature was 36.8°C, pulse rate 86 beats/min, respiratory rate 18 breaths/min, and blood pressure 158/86 mm Hg. He was alert and in fair general condition. There was no facial edema. Lung sounds were clear, and no dry or moist rales were heard. His heart rhythm was regular without pathological murmurs. The abdomen was soft without tenderness or rebound tenderness. No renal percussion tenderness or lower-limb edema was present. Gastrocnemius tenderness was not obvious.

### 2.2. Diagnostic assessment

Early laboratory tests after admission indicated severe renal dysfunction, metabolic acidosis, and marked muscle enzyme elevation. Serum urea was 38.36 mmol/L, serum creatinine 1202 μmol/L, blood urea nitrogen (BUN)/creatinine ratio approximately 7.9, and cystatin C 4.91 mg/L. CK was 28,831 U/L, myoglobin 915 ng/mL, creatine kinase-MB isoenzyme (CK-MB) 183.83 ng/mL, lactate dehydrogenase 1049 U/L, and α-hydroxybutyrate dehydrogenase 811 U/L. Alanine aminotransferase (ALT) was 209 U/L, and aspartate aminotransferase (AST) was 532 U/L. Serum potassium was 4.63 mmol/L (5.56 mmol/L at the outpatient visit), calcium 1.78 mmol/L, and phosphorus 2.09 mmol/L. Arterial blood gas analysis showed pH 7.27, partial pressure of carbon dioxide 26.2 mm Hg, bicarbonate 11.7 mmol/L, and standard base excess − 14.1 mmol/L. Urinalysis showed glucose 3+, protein 3+, urine osmolality 373 mOsm/kg, specific gravity 1.011, pH 6.0, occult blood 3+, and red blood cells 16/μL. Urinary albumin was 197 mg/L, and the urinary albumin-to-creatinine ratio was 345 mg/g.

Autoimmune tests showed antinuclear antibody positivity at 1:320, exceeding the laboratory reference threshold for negativity (<1:100), and weak positivity for anti-nucleosome antibody. Anti-double-stranded DNA, anti-Sm, anti-Jo-1, anti-SSA/SSB, anti-glomerular basement membrane, and vasculitis-associated antibodies were negative. Thyroid function, tumor markers, immunoglobulin G4, and complement levels did not support a specific alternative diagnosis. Chest computed tomography showed bilateral bronchial lesions, scattered tiny pulmonary nodules, and fibrous proliferative changes in the right upper lobe. Echocardiography showed mild mitral, tricuspid, and aortic regurgitation. Urinary ultrasonography showed status after right nephrectomy, abnormal echogenicity and a cyst in the left kidney, and no left renal artery or vein stenosis or thrombosis (Table 2).

**Table 2:** Main laboratory changes during hospitalization.

Time point	eGFR (mL/min/1.73 m^2^)	Scr (μmol/L)	Urea (mmol/L)	CK (U/L)	Myo (ng/mL)	K (mmol/L)	Ca/P (mmol/L)	ALT/AST (U/L)	Urine protein	Urine glucose
>2 mo before admission	43.7	134	7.68	Not tested	Not tested	4.88	2.24/1.05	32/32	+	−
Outpatient/admission day	Not tested	1132	35.73	Not tested	Not tested	5.56	2.01/2.30	Not tested	3+	3+
Hospital day 2	3.1	1202	38.36	28,831	915	4.63	1.78/2.09	209/532	3+	3+
Hospital day 7	3.0	1218	35.92	11,194	891	4.16	1.72/2.43	211/226	2+	2+
Hospital day 15	Not tested	733	19.80	228	357	3.92	1.93/1.88	76/70	2+	2+
Hospital day 20 (3 d before discharge)	7.1	694	21.38	98	245	3.59	1.99/2.06	59/60	2+	+

ALT = alanine aminotransferase, AST = aspartate aminotransferase, CK = creatine kinase, eGFR = estimated glomerular filtration rate, Myo = myoglobin, Scr = serum creatinine.

The patient lacked typical myalgia, muscle weakness, or dark-colored urine; however, the marked CK elevation, elevated myoglobin, strong urine occult blood with few red blood cells, AST-predominant transaminase elevation, hypocalcemia, hyperphosphatemia, and metabolic acidosis were consistent with RM. According to the Kidney Disease: Improving Global Outcomes (KDIGO) criteria, the substantial serum creatinine increase from baseline and the subsequent requirement for renal replacement therapy were consistent with KDIGO stage 3 AKI superimposed on CKD.

The patient developed elevated muscle enzymes and a rapid rise in serum creatinine after approximately 2 months of fenofibrate exposure. CK decreased rapidly after fenofibrate withdrawal, and common causes such as heat stroke, seizure, crush injury, strenuous exercise, and severe infection or intoxication were not identified. Therefore, fenofibrate-associated RM was considered probable.

Differential diagnoses included acute myocardial infarction, inflammatory myopathy, chronic hepatitis B activity or drug-induced liver injury, tenofovir-related proximal tubular injury, and prerenal AKI. Acute myocardial infarction was considered unlikely because the patient had no chest pain, electrocardiography did not support myocardial infarction, and the degree of CK elevation was more consistent with skeletal muscle injury. Inflammatory myopathy was not supported by the absence of proximal muscle weakness, negative typical myositis antibodies, and rapid muscle enzyme decline after drug withdrawal and supportive treatment. Chronic hepatitis B activity was unlikely because hepatitis B virus DNA was within the normal range, and the pattern of aminotransferase elevation, with markedly elevated CK and lactate dehydrogenase, suggested muscle injury as the main source, although follow-up of liver function was still required. Tenofovir-related proximal tubular injury was suspected given the presence of 3+ glucosuria without concomitant hyperglycemia. However, isolated proximal tubular dysfunction could not account for the markedly elevated creatine kinase level of 28,831 U/L and increased myoglobin levels. Furthermore, repeated urinalyses performed prior to admission during tenofovir therapy had not revealed glucosuria. Prerenal AKI was also considered because of poor intake and vomiting, but urine osmolality, the urea/creatinine ratio, and clinical features did not support isolated prerenal AKI.

### 2.3. Therapeutic interventions

After admission, the patient was initially managed as having severe AKI superimposed on CKD with metabolic disturbances. Once marked CK and myoglobin elevation were identified, fenofibrate was discontinued immediately. Fluid therapy, correction of metabolic acidosis, urine output and electrolyte monitoring, and kidney-protective supportive treatment were initiated. Because hyperkalemia had been present before admission and the patient initially refused dialysis, sodium zirconium cyclosilicate was used early during hospitalization to control serum potassium, with dose adjustment according to serial potassium measurements. Potassium-wasting diuretics were avoided during the period in which volume depletion was possible.

On hospital days 4 to 7, metabolic acidosis partially improved, but serum creatinine remained approximately 1200 μmol/L, myoglobin remained elevated, and uremic gastrointestinal symptoms such as nausea, vomiting, and poor appetite persisted. After repeated discussions with the patient, IHD was started on hospital day 10, 3 times weekly. Because the patient had no clinical evidence of fluid overload and remained at risk of volume depletion, net ultrafiltration was set at 0 mL to preserve renal perfusion and support kidney recovery. The purpose of IHD was not the direct removal of fenofibrate or myoglobin but supportive treatment for persistent uremic symptoms, severe AKI, and metabolic acidosis. After hemodialysis, gastrointestinal symptoms gradually improved and urine output increased.

### 2.4. Follow-up and outcomes

The patient was discharged after 23 days of hospitalization. As renal function recovered, the dialysis frequency was gradually reduced from twice weekly to once weekly and eventually stopped (Table 3). At 8 months after discharge, serum creatinine had further decreased to 157 μmol/L, and myoglobin and CK remained within the normal range.

**Table 3:** Follow-up laboratory findings and dialysis adjustment after discharge.

Follow-up time	eGFR (mL/min/1.73 m^2^)	Scr (μmol/L)	Urea (mmol/L)	CK (U/L)	Myo (ng/mL)	K (mmol/L)	Ca/P (mmol/L)	Urine protein	Urine glucose	Dialysis status
1 wk after discharge	10.5	497	13.03	Not tested	Not tested	3.26	2.11/0.86	Not tested	Not tested	Reduced to twice weekly
2 wk after discharge	12.4	429	11.68	Not tested	Not tested	3.63	1.91/0.60	2+	−	Reduced to once weekly
4 wk after discharge	18.1	311	12.25	Not tested	Not tested	3.69	2.01/0.62	Not tested	Not tested	Gradually reduced
5 wk after discharge	18.1	310	17.15	72	132	3.93	2.13/0.82	2+	−	Hemodialysis discontinued
3 mo after discharge	29.3	204	8.91	Not tested	Not tested	4.42	2.28/1.12	Not tested	Not tested	Hemodialysis discontinued
8 mo after discharge	39.7	157	8.49	68	82	4.32	2.13/0.84	+	−	Hemodialysis discontinued

During maintenance hemodialysis, laboratory tests were performed before dialysis.

## 3. Discussion

Fenofibrate improves triglyceride metabolism by activating peroxisome proliferator-activated receptor alpha and has established therapeutic value in severe hypertriglyceridemia and mixed dyslipidemia. Fenofibrate-associated rhabdomyolysis (RM) is rare but can cause severe acute kidney injury (AKI) and may be life-threatening.^[4]^ To compare this case with previous reports, we conducted a focused narrative review of the English-language literature published through May 2026. Relevant reports were identified using combinations of the terms “fenofibrate,” “rhabdomyolysis,” “myopathy,” “acute kidney injury,” and “acute renal failure,” supplemented by manual screening of the reference lists of relevant case reports and reviews. Thirteen reports describing 14 patients were included.^[5-17]^ Published cases indicate that fenofibrate-associated RM may occur during either monotherapy or concomitant statin therapy. Some patients progressed to severe AKI requiring kidney replacement therapy, although varying degrees of kidney recovery were generally observed after drug withdrawal and supportive care. Reported predisposing factors included renal impairment, hypothyroidism, diabetes mellitus, older age, and polypharmacy.^[5-17]^ Compared with previous reports, the main distinguishing feature of this case was the absence of typical myalgia, muscle weakness, and dark-colored urine despite progression to KDIGO stage 3 AKI requiring dialysis, followed by recovery to dialysis independence (Table 4). Because the classic triad of RM is uncommon in clinical practice, nonspecific presentations may lead to missed or delayed diagnosis.^[18]^

**Table 4:** Published cases of fenofibrate-associated rhabdomyolysis and comparison with the present case.

Reference	Drug exposure	Major risk factors/underlying diseases	Presentation and key laboratory findings	Renal injury/RRT	Outcome and relevance to the present case
Satarasinghe et al, 2007^[5]^	Fenofibrate monotherapy	Unrecognized hypothyroidism	Myopathy/RM; reported dialysis-dependent acute renal failure	Dialysis-requiring ARF	Suggests that hypothyroidism can markedly increase fenofibrate-related myotoxicity; similar to our case in severe AKI and dialysis requirement
Tahmaz et al, 2007^[6]^	Fenofibrate monotherapy for approximately 4 weeks	Hypertension and dyslipidemia; no known diabetes, hypothyroidism, or CKD	Generalized myalgia; CK approximately 21,000 U/L	SCr approximately 5.5 mg/dL; ARF without dialysis	Recovered after drug withdrawal and supportive therapy; indicates that RM-AKI may occur even without classic risk factors
Unal et al, 2008^[7]^	Fenofibrate plus statin; 2 cases	Coronary artery disease and combination lipid-lowering therapy	Severe myalgia, hemoglobinuria, oliguria; CK approximately 97,000 U/L	ARF in both cases; both received HD	Recovered after drug withdrawal, fluids, alkalinization/mannitol, and HD; illustrates increased risk with statin-fibrate combination therapy
de Sousa et al, 2009^[8]^	Fenofibrate monotherapy	CKD stage 3 and hypothyroidism	RM with abnormal CK and renal function	AKI/renal deterioration	Emphasizes screening for thyroid dysfunction and CKD-related risk before treatment
Danis et al, 2010^[9]^	Fenofibrate therapy	Diabetes mellitus and dyslipidemia	Generalized myalgia, fatigue, oliguria	ARF	Improved after drug withdrawal, fluids, and urinary alkalinization; supports the importance of early monitoring
Cansu et al, 2011^[10]^	Fenofibrate monotherapy for approximately 1 month	Diabetes mellitus and hypertension	Fatigue and myalgia; CK approximately 11,030 U/L	SCr approximately 6.9 mg/dL; ARF without dialysis	Recovered after drug withdrawal, fluids, and alkalinization; similar to our case in AKI after monotherapy exposure
Kato et al, 2011^[11]^	Fenofibrate monotherapy	Advanced age and chronic myelogenous leukemia	Generalized myalgia and right abdominal pain with psoas involvement; CK approximately 5882 U/L	SCr approximately 4.1 mg/dL	Suggests that manifestations may be focal and atypical; our case mainly presented with gastrointestinal symptoms
Soyoral et al, 2012^[12]^	Fenofibrate monotherapy	Type 2 diabetes mellitus, CKD stage 4, and heart failure	Fatigue and diffuse myalgia; CK approximately 13,000 U/L	SCr approximately 8.4 mg/dL; no dialysis	Highlights the need for dose adjustment and close follow-up in chronic renal impairment
Erdur et al, 2012^[13]^	Fenofibrate monotherapy	Nephrotic syndrome, chronic renal failure, and hypoalbuminemia	Fatigue and severe myalgia; CK approximately 28,261 U/L	Renal dysfunction without dialysis	Suggests that hypoalbuminemia and nephrotic syndrome may increase drug exposure and myotoxicity
Kiskac et al, 2013^[14]^	Fenofibrate restarted after 1 month off therapy	Hypertension and dyslipidemia	Muscle weakness, fatigue, and dark urine; CK approximately 9627 U/L	SCr approximately 1.49 mg/dL; ARF without dialysis	Re-exposure supports drug relatedness; no rechallenge was performed in our case, so causality should be described as suspected/probable
Marques et al, 2014^[15]^	Fenofibrate monotherapy	Adolescent, chronic renal failure, severe TG elevation, and hypothyroidism	RM followed by irreversible renal deterioration	Required HD and later kidney transplantation	Suggests that CKD plus hypothyroidism may lead to poor renal outcomes; our patient required HD but ultimately discontinued dialysis
Wang et al, 2018^[16]^	Fenofibrate monotherapy	Hypothyroidism and female sex	RM; CK approximately 9890 U/L	SCr approximately 140 μmol/L; no dialysis	Improved after drug withdrawal, fluids, diuresis, and alkalinization; emphasizes thyroid function monitoring
Zhou et al, 2020^[17]^	Micronized fenofibrate for approximately 5 mo	Post-pancreatitis diabetes mellitus	Fatigue, myalgia, and decreased urine output; CK 8385 U/L; myoglobin 1075 ng/mL	SCr 97.63 μmol/L; elevated BUN; no dialysis	Recovered after drug withdrawal, fluids, alkalinization, and diuresis; shows that monotherapy-associated RM may occur in diabetes-related metabolic disorders
Present case	Fenofibrate 0.2 g/day for approximately 2 mo	70-yr-old man, CKD, solitary kidney, and polypharmacy	Nausea, anorexia, and rapid SCr rise without typical myalgia or dark urine; CK 28,831 U/L; myoglobin 915 ng/mL	SCr 1202 μmol/L; KDIGO stage 3 AKI; short-term HD	Distinctive features include atypical presentation, solitary kidney with CKD, dialysis-requiring AKI, and subsequent renal recovery with dialysis independence

Some laboratory values are retained in the original units reported by the cited articles. This table summarizes the published cases included in the focused narrative review and does not represent a systematic review or meta-analysis.

AKI = acute kidney injury, ARF = acute renal failure, BUN = blood urea nitrogen, CK = creatine kinase, CKD = chronic kidney disease, HD = hemodialysis, RM = rhabdomyolysis, RRT = renal replacement therapy, SCr = serum creatinine, TG = triglyceride.

RM-associated AKI is driven by direct tubular toxicity and oxidative injury from myoglobin, intratubular cast formation and obstruction caused by the interaction of myoglobin with Tamm–Horsfall protein in an acidic urinary environment, and intrarenal vasoconstriction resulting from reduced effective circulating volume.^[1,2]^ Accordingly, management centers on prompt removal of the precipitating factor, maintenance of adequate effective circulating volume, and correction of acid–base and electrolyte disturbances. Avoiding volume depletion is particularly important because further reductions in renal perfusion and tubular flow may promote myoglobin concentration, cast formation, and tubular injury. Fluid therapy should nevertheless be individualized according to urine output, cardiac function, and volume status to avoid fluid overload in patients with oliguria or anuria. Current evidence does not support the routine use of urinary alkalinization, diuretics, or mannitol to prevent RM-associated AKI.^[19]^ Kidney replacement therapy should be initiated for conventional AKI indications, such as refractory metabolic acidosis, hyperkalemia, fluid overload, or uremic symptoms, rather than solely on the basis of the myoglobin level.^[20]^ Conventional hemodialysis is primarily used to manage AKI-related complications rather than to directly remove myoglobin. Fenofibric acid, the active metabolite of fenofibrate, is highly protein-bound; therefore, hemodialysis should not be considered a principal means of drug removal.^[21]^ Although newer dialysis membranes, hemoadsorption, and sequential extracorporeal blood purification techniques may enhance the removal of myoglobin or inflammatory mediators, evidence that they improve patient-important outcomes, such as kidney recovery, freedom from dialysis, or survival, remains insufficient.^[22]^

Electrolyte abnormalities in RM are phase-dependent. Early muscle injury can cause hyperkalemia, hyperphosphatemia, and hypocalcemia through intracellular electrolyte release, calcium influx into injured myocytes, and calcium–phosphate deposition in necrotic muscle.^[1,2]^ Hyperkalemia requires prompt recognition and treatment because of the risk of life-threatening arrhythmia. Routine calcium replacement is generally unnecessary for early asymptomatic hypocalcemia and should be reserved for symptoms or for membrane stabilization during severe hyperkalemia. During recovery, mobilization of calcium from injured tissues may cause rebound hypercalcemia; therefore, potassium, phosphate, and calcium should be monitored during both the acute and recovery phases.^[2]^

Several limitations affect causal attribution in this report. Because a kidney biopsy was not performed, myoglobin cast nephropathy could not be confirmed histopathologically. Urinary myoglobin and a comprehensive panel of markers of proximal tubular function were not measured; therefore, concomitant tenofovir-associated proximal tubular injury or Fanconi syndrome could not be fully excluded.^[23]^ For safety and ethical reasons, fenofibrate was not reintroduced for a rechallenge, precluding classification of the causal relationship as definite. Nevertheless, the temporal relationship between drug exposure and disease onset, the positive dechallenge reflected by declining muscle enzyme levels and clinical improvement after drug withdrawal, and the absence of a more plausible common precipitant support fenofibrate-associated RM as an important contributor to this episode of severe AKI.

Before fenofibrate is prescribed to older adults or patients with CKD or limited renal functional reserve, eGFR should be assessed and the dose adjusted – or treatment avoided – according to the prescribing information for the specific formulation. Kidney function should be reassessed early after treatment initiation or dose adjustment.^[24,25]^ If a patient develops unexplained deterioration in kidney function, gastrointestinal symptoms, elevated aspartate aminotransferase levels, or a positive urine dipstick for blood with few red blood cells on urine sediment after initiation or modification of lipid-lowering therapy, serum creatine kinase and myoglobin levels should be measured promptly to reduce the risk of missed or delayed diagnosis of RM.^[18]^

## 4. Conclusion

Fenofibrate-associated rhabdomyolysis is uncommon but may cause severe, dialysis-requiring AKI, and its nonspecific clinical manifestations may delay diagnosis. Prompt recognition and withdrawal of the suspected offending drug, supportive management, and appropriate initiation of kidney replacement therapy are essential for promoting kidney function recovery.

## Author contributions

**Writing – original draft:** Yumei Fang.

**Writing – review & editing:** Yan Jiang.
